# Abiotic and biotic correlates of the occurrence, extent and cover of invasive aquatic *Elodea nuttallii*

**DOI:** 10.1111/fwb.13960

**Published:** 2022-07-01

**Authors:** Kate Crane, Louise Kregting, Neil E. Coughlan, Ross N. Cuthbert, Anthony Ricciardi, Hugh J. MacIsaac, Jaimie T.A. Dick, Neil Reid

**Affiliations:** ^1^ School of Biological Sciences Queen’s University Belfast Belfast UK; ^2^ Queen’s University Marine Laboratory Portaferry UK; ^3^ School of Natural and Built Environment Queen’s University Belfast Belfast UK; ^4^ School of Biological, Earth and Environmental Science University College Cork Cork Ireland; ^5^ GEOMAR Helmholtz‐Zentrum für Ozeanforschung Kiel Kiel Germany; ^6^ Redpath Museum McGill University Montreal QC Canada; ^7^ Great Lakes Institute for Environmental Research University of Windsor Windsor ON Canada; ^8^ Institute for Global Food Security (IGFS) Queen’s University Belfast Belfast UK

**Keywords:** competition, *Dreissena polymorpha*, freshwater ecosystems, macrophytes, nutrients

## Abstract

1. Biological invasions, especially invasive alien aquatic plants, are a major and growing ecological and socioeconomic problem worldwide. Freshwater systems are particularly vulnerable to invasion, where impacts of invasive alien species can damage ecological structure and function. Identifying abiotic and biotic factors that mediate successful invasions is a management priority. Our aim was to determine the environmental correlates of *Elodea nuttallii*; a globally significant invasive aquatic species.
2. *Elodea nuttallii* presence/absence (occurrence), extent (patch area) and percentage cover (density) was visually assessed from a boat throughout Lough Erne (approximately 144 km^2^), County Fermanagh, Northern Ireland during the active summer growth season (July–September). In addition, substrate type and zebra mussel *Dreissena polymorpha* occurrence was recorded. Fourteen water chemistry variables were collected monthly from 12 recording stations throughout the lake during the 9 years before the survey to spatially interpolate values and establish temporal trajectories in their change. Shoreline land use was derived from CORINE land cover maps. Environmental associations between *E. nuttallii*, substrate, *D. polymorpha*, water chemistry and land use were assessed.
3. *Elodea nuttallii* occurrence was positively associated with water conductivity, alkalinity, suspended solids, phosphorus (both total and soluble) and chlorophyll‐*a* concentrations, but negatively associated with pH and total oxidised nitrogen. *E. nuttallii* patch extent and proportional cover were positively associated, to varying degrees, with the presence of *D. polymorpha*, biological oxygen demand, water clarity and soft substrate, but negatively associated with urban development and ammonium.
4. *Elodea nuttallii* displayed high levels of phenotypic plasticity in response to environmental variation, allowing it to adapt to a wide range of conditions and potentially gain competitive advantage over native or other invasive macrophytes.
5. It is evident that multiple abiotic and biotic factors, including facilitation by co‐occurring invasive dreissenid mussels, interact to influence the distribution and abundance of *E. nuttallii*. Thus, it is necessary to consider a more comprehensive environmental context when planning *Elodea* management strategies.

## 1 INTRODUCTION

Biological invasions continue to alter and, in some cases, detrimentally impact ecosystem function worldwide, resulting in a plethora of environmental, economic and social problems (Haubrock et al., 2021; Simberloff et al., 2013). Rates of biological invasion continue to increase, with intensifying trade and transport networks driving introductions from disparate biogeographical regions (Bailey et al., 2020; Seebens et al., 2017, 2021). Management strategies for invasive alien species are reliant on evidence‐based, data‐driven science (Dick et al., 2017), yet have hitherto been largely insufficient in reducing ecological and economic damages (Cuthbert, Pattison, et al., 2021a). Specifically, understanding the abiotic and biotic correlates of biological invasions, whose status can be quantified using numerous metrics such as occurrence, extent or proportional cover, requires a holistic approach in considering potential drivers of invasion throughout the total environment. Therefore, the consideration of multiple variables in concert is increasingly required to better understand and predict invasions worldwide.

Aquatic ecosystems are regarded as particularly vulnerable to biological invasions and their impacts (Ricciardi & MacIsaac, 2011). Invasive aquatic macrophytes often are recorded to be ecologically and economically damaging, for example, through increased flood risk, devaluation of property, the disruption of navigation, water abstraction, irrigation and recreational activities (Hussner et al., 2017; Oreska & Aldridge, 2011). Studies typically have focused on a small number of variables to explain invasion success, including the relationships between macrophytes and spatiotemporal patterns in water quality and the surrounding land use (Lougheed et al., 2001; Sass et al., 2010). A more comprehensive understanding of invasion drivers, including the emergent effects of other species impacts and their abiotic interactions, is needed to enhance predictive capacities for future invasions.

*Elodea nuttallii* (Planch.) St John is a perennial, submerged aquatic macrophyte which is native to North America and invasive in Europe, Asia and Australasia (Barrat‐Segretain et al., 2002; Jones et al., 2000; Zehnsdorf et al., 2015). Introduced to Great Britain and Ireland in 1966, *E. nuttallii* typically inhabits lakes, ponds and slow‐moving rivers (Barrat‐Segretain et al., 2002; Champion et al., 2010), often displacing *Elodea canadensis*, a congeneric invasive macrophyte (Simpson, 1990). Notably, *E. nuttallii* can grow in highly eutrophic, turbid waters (Cook & Urmi‐König, 1985; Ozimek et al., 1993; Thiébaut & Muller, 1999), and can rapidly dominate invaded ecosystems through the formation of dense monospecific stands that outcompete native macrophytes and significantly alter freshwater communities (Angelstein & Schubert, 2008; Champion et al., 2010; Thouvenot & Thiébaut 2018; Zehnsdorf et al., 2015). Furthermore, it appears that *E. nuttallii* can be transported long distances overland by anthropogenic vectors (Coughlan et al., 2018; Kelly et al., 2014).

Interestingly, *E. nuttallii* often co‐occurs with invasive dreissenid bivalves (Crane et al., 2020), such as the zebra mussel *Dreissena polymorpha* (Pallas 1771), itself a prolific Ponto‐Caspian species that frequently dominates invaded ecosystems, causing myriad ecological and socioeconomic impacts (Sousa et al., 2014). *Dreissena polymorpha* impacts include displacement of native mussels, increased water clarity, altered nutrient cycling, and shifts in filamentous algal blooms and macrophyte assemblage composition (Ricciardi, 2003; Ricciardi et al., 1998; Rosell et al., 1999; Ward & Ricciardi, 2007). Given increasing accumulations of invaders in aquatic ecosystems (Ricciardi, 2015), understanding how multiple invasive alien species interact and respond to variability in environmental conditions is essential to understanding the spatiotemporal dynamics of invasions, which inform the scale of any management actions required. Empirical data indicate that the presence of *D. polymorpha* can promote facilitative interactions in favour of *E. nuttallii* (Crane et al., 2020). However, the extent of potential facilitations between invasive alien species in field‐based conditions has not been rigorously tested, nor compared with additional environmental variables that might mediate invasion dynamics.

The aim of this study was to identify the environmental correlates of a major *E. nuttallii* invasion in Lough Erne; a large (c. 144 km^2^) freshwater lake in Northern Ireland, capturing the invasion status using three metrics: occurrence (presence/absence), extent (area in hectares) and proportional cover (percentage cover). The objective was to assess environmental correlates of *E. nuttallii* including spatial and temporal trends in water quality variables, as well as surrounding land cover and land use. In addition, we included the co‐occurrence of *E. nuttallii* with *D. polymorpha* within our analyses with the objective of assessing the potential role played by interspecific interactions in the distribution of *E. nuttallii* alongside the environmental variation.

## 2 METHODS

### 2.1 Study site

We assessed environmental correlates of the *E. nuttallii* invasion of Lough Erne, County Fermanagh, Northern Ireland, UK. *Elodea nuttallii* was first recorded in Lough Erne in 2006, and by 2010 it had spread extensively, causing significant ecological, navigational and recreational problems, while competitively displacing *E. canadensis* that has invaded previously (Kelly et al., 2015). Lough Erne is a naturally eutrophic lake consisting of two major waterbodies connected by a 10‐km stretch of the River Erne (O’Higgins, 2018). Lower Lough Erne (109 km^2^) is the third largest lake in the UK, with a mean depth of 11.9 m. Upper Lough Erne (34.5 km^2^) has a mean depth of 2.3 m (Lawrie et al., 1992) and comprises an array of wide river channels, open lake and numerous islands, creating a highly complex sinuous shoreline. The water chemistry of the two lakes reflects the underlying geology of limestone and sandstone which gives rise to both carbonate‐rich and softly acidic waters. The catchment has a sparse human population and is largely agricultural, with mature, semi‐natural riparian woodland. Designated as a Ramsar Site (DOE, 1997), Special Protection Area (DOE, 1997) and including ten Areas of Special Scientific Interest (DAERA, 2018), Upper Lough Erne is further classified as a Special Area of Conservation (JNCC, 2015), and the Lough is an internationally important area of conservation concern.

### 2.2 Surveys

Upper and Lower Lough Erne were circumnavigated by boat and a visual distribution survey was carried out for mature *E. nuttallii* at the peak of the growing season, July–September 2015. Immature plants at depth can be surveyed using rake samples, but this was not undertaken here as a consequence of the near ubiquity of obvious and mature patches of *Elodea* within the study system. High water clarity allowed for the visual detection of stands/swards of plants. A bathyscope and waterproof GoPro Hero 5 were used to identify the extent of each patch of macrophytes. The boundary of each patch was recorded using a handheld Trimble Yuma Rugged GPS unit (model YMA‐FGS6AS‐00) and mapped using ARCMAP 10.5 (ESRI, California, USA). Multiple GPS points were used to ensure that the boundaries of patches were recorded accurately. The proportional cover of *E. nuttallii* relative to other vegetation within the patch was visually estimated by eye using an ordinal categorical scale: “0” = 0% *E. nuttallii*, 100% native macrophytes; “1” = 0%–25% *E. nuttallii*; *“*2” = 26%–50% *E. nuttallii*; “3” = 51%–75% *E. nuttallii*; “4” = 76%–100% *E. nuttallii*.

### 2.3 Environmental variables

During the surveys, substrate type was visually estimated as being either hard (fine and coarse gravel/shingle, and larger stones and/or rock) or soft (silt, sand and mud). *Dreissena polymorpha* presence or absence (also visually estimated) was recorded for each vegetation patch. Other invasive bivalves are not known to reside within the study site, and there is a paucity of native bivalves. Presence was recorded on a binary scale, as visual survey from a boat precluded robust determination of abundance. For logistical and practical purposes, substrate samples as well as water quality measures were not measured empirically at each surveyed macrophyte patch. Instead, 14 water quality variables were obtained from the Northern Ireland Environment Agency (NIEA) Water Management Unit, who have carried out routine, standardised, lake water monitoring and surveillance since 2006 in compliance with the Water Framework Directive, National Water Quality Monitoring Programme (Table S1). Water samples were collected monthly by NIEA staff from 12 recording stations throughout the lake system (Figure S1). Recording stations were c. 5 km apart, much further than the distance between surveyed macrophyte patches. Thus, the 14 water chemistry variables were Kriged using the Spatial Analyst toolbox in ArcGIS to interpolate values for regions between recording stations, and to extrapolate values out to the lake’s edge to create a heat map of spatial variation in each variables (Figure S1). Kriging used an ordinary spherical semivariogram model with a search radius of three points; as recording stations were distributed linearly from the lake’s source to mouth, this emulated a moving window averaging water chemistry values across a combination of three stations (one upstream, one in the middle, one downstream), providing ecologically plausible smoothed heat map surfaces for each variable (Figure S1). In addition to spatial interpolation, temporal trends in water chemistry variables also were captured. Data for each variable were plotted separately against time for each recording station, and a linear regression fitted to obtain the regression coefficient, i.e. the slope of the line (*β*‐value) representing the direction of any trend in that variable at each station. Raw data were standardised to have a *x̄* = 0 and *σ* = 1 so that regression coefficients were directly comparable. For each variable, spatiotemporal variation was again captured by Kriging the spatially explicit regression coefficients as above to create a heat map of change in each variable from 2006 to 2015 (Figure S1). Imputed values for both the mean and regression coefficient for each variable were extracted from the heat map surfaces for the centroid of each macrophyte patch of *E. nuttallii* mapped during the survey. It should be noted that variation in water chemistry values between adjacent macrophyte patches was, necessarily, minimised by the nature of the interpolation methods which is further commented on in the Discussion.

Land cover and land use were derived from the Land Cover Map (2007), produced by the Centre for Ecology and Hydrology from analysis of satellite or aerial imagery (www.ceh.ac.uk/services/land‐cover‐map‐2007). Each patch of *E. nuttallii* was buffered to a distance of 500 m, and the extent (area in hectares) of terrestrial land cover or land use within each was extracted representing variation in adjacent habitats. In total, 31 environmental variables were measured or estimated for each patch of *E. nuttallii* (Table 1).

**TABLE 1:** Principal components analysis (PCA) of environmental variables describing water quality, trends in water quality and land cover/use.

Variable groupings		Principal component axes					
		PC1	PC2	PC3	PC4	PC5	PC6
	Eigenvalue	10.277	4.490	4.152	3.066	1.718	1.640
	% variance	33.150	14.484	13.395	9.892	5.541	5.291
	Cumulative % variance	33.150	47.635	61.082	70.920	76.461	81.752
Water quality, Mean values (2006–15)	1. pH	**−0.926**	0.227	−0.196	0.096	−0.132	0.091
	2. ALK	**0.804**	0.318	0.121	0.304	−0.069	−0.034
	3. COND	**0.935**	0.093	−0.144	0.208	0.084	0.144
	4. COL	−0.226	−0.254	**0.789**	0.352	−0.036	−0.293
	5. SS	**0.954**	0.098	−0.005	0.197	0.087	0.049
	6. P(SOL)	**0.878**	−0.267	0.221	0.141	0.099	−0.054
	7. P(TOT)	**0.755**	−0.530	−0.040	0.166	0.123	0.072
	8. NH_4_	0.299	0.178	**0.862**	0.223	0.060	−0.187
	9. NO_2_	0.299	−0.051	−0.032	0.394	**−0.504**	−0.357
	10. TOxN	**−0.926**	0.267	−0.064	−0.137	−0.070	0.106
	11. CHL *A*	**0.939**	−0.231	−0.211	−0.009	0.096	0.059
	12. BOD	−0.195	−0.269	**−0.753**	−0.319	−0.046	0.263
	13. pH	−0.024	**−0.886**	−0.183	−0.119	0.036	−0.083
Temporal trends (2006–15)	14. ALK	0.263	**−0.651**	0.166	**0.642**	0.126	0.098
	15. COND	0.479	0.015	0.103	**0.755**	0.085	0.111
	16. COL	−0.203	**0.877**	0.007	−0.329	−0.007	−0.032
	17. SS	−0.188	**0.849**	0.258	0.262	−0.043	0.078
	18. P(SOL)	**−0.890**	0.403	0.157	−0.008	−0.092	−0.033
	19. P(TOT)	0.013	0.448	0.486	0.038	0.359	0.110
	20. NH_4_	**−0.528**	0.117	**−0.601**	**−0.529**	−0.106	0.038
	21. NO_2_	−0.211	−0.307	−0.343	**−0.773**	0.151	0.095
	22. TOxN	**0.915**	−0.121	0.212	0.250	0.042	−0.119
	23. CHL A	0.413	**−0.585**	0.018	−0.207	0.014	0.246
	24. BOD	**0.836**	0.222	0.395	0.221	0.130	−0.022
Land cover/use	25. Other farmland	0.264	−0.173	−0.258	−0.156	**0.650**	−0.207
	26. Improved grassland	0.071	0.043	0.107	0.005	0.282	**−0.869**
	27. Conifer plantations	−0.126	−0.010	−0.129	−0.212	**−0.587**	0.108
	28. Deciduous woodland	0.327	−0.007	−0.410	0.276	−0.135	0.445
	29. Other woody habitats	−0.479	0.185	−0.198	−0.108	−0.171	0.288
	30. Bog, fen & moor	0.117	−0.015	0.067	−0.031	**0.520**	−0.090
	31. Urban/suburban areas	0.038	0.171	**0.812**	−0.143	−0.010	0.183

Bold values indicate the presence of substantial principle component axis loadings (*r* > 0.5)

### 2.4 Data analyses

Principal components analysis (PCA) with varimax rotation was used to reduce an initially large set of putative explanatory variables, exhibiting a high degree of multicollinearity, to a much‐reduced set. Statistical and ecological convention is that all principal component axes with an eigenvalue >1.0 are retained for further analyses (following, e.g., Kaiser, 1960, Franklin et al., 1995 and Björklund, 2019).

Generalised linear mixed models (GLMMs) were used to examine three dependent variables: *E. nuttallii* (i) occurrence was fitted as a binomial distribution with a logit link function (accounting for its binary [0/1] distribution); (ii) extent, i.e. area in hectares fitted as a Gamma distribution with a logit link function (accounting for its positive right‐skew, i.e., a high frequency of small patches and a low frequency of large patches); and (iii) proportional cover, i.e. estimated percentage cover within each patch fitted using a Gaussian distribution and identity link function (accounting for its normal bell‐curve distribution). Model structure was identical for all three models: Lough_ID (i.e., Upper or Lower) was fitted as a two‐level random factor to account for the pseudoreplication of multiple observations per lake whilst *D. polymorpha* presence or absence (0/1) and substrate type (hard or soft) were fitted as two‐level fixed factors. Six principal component axes representing the water quality variables and land cover or land use were fitted as covariates. The residuals of each model were examined using Kolmogorov–Smirnov tests to ensure normality and adherence to the assumptions of each test and its selected distribution. Models were used to assess the environmental correlates *E. nuttallii* and were not used for prediction, i.e. there was no creation of probability heat surfaces or extrapolation of values to unsurveyed sites. For this reason, the information theoretic approach of all subset regression with model selection using the Akaike Information Criterion (AIC) was not used. Instead, fitting the global model was favoured for the purposes of comparability, i.e. fitting a standard model structure for each independent variable allowing the effects of each variable to be explicitly reported, even at *p* > 0.05.

Each principal component axis was associated with a large number of collinear variables and not all may have been related to *E. nuttallii*. However, a variety of individual environmental variables associated with substantial principal component axis loadings (*r* > 0.5; Table 1) were found to be significantly associated (*p* < 0.05) with each of the three dependent variables across different model axes using GLMM (i.e., PC1 for binary *E. nuttallii* occurrence and PC3 for *E. nuttallii* area and proportional cover; Table 2). Most environmental variables were nonparametric. Thus, Mann–Whitney *U*‐tests were used to determine significant differences in PC1 variables between sites where *E. nuttallii* was present compared to those where it was absent. Spearman’s rank correlations were used to determine significant relationships between PC3 variables and *E. nuttallii* extent (patch size) and Kruskal–Wallis tests were used to determine significant relationships between PC3 variables and *E. nuttallii* proportional cover category. Probability values from multiple tests were Bonferroni‐corrected for multiple comparisons. Statistical analyses were performed using SPSS v25 (IBM) and all significant relationships were plotted using SIGMAPLOT v12 (Systat Software Inc.).

**TABLE 2:** Generalised linear mixed model (GLMM) results for *Elodea nuttallii* (a) occurrence, (b) extent (i.e., area in ha) and (c) proportional cover (i.e., % cover where lough (Upper and Lower) was fitted as a random factor

Model; Explanatory variables	*β* ± *SE*	*F*	*n.df*.	*d.df*.	*p*
(a) *E. nuttallii* presence (*F*_9,195_ = 1.017, *p* = 0.428, AUC = 0.750)					
Depth	−0.075 ± 0.239	0.098	1	195	0.755
*D. polymorpha* presence	−0.154 ± 0.400	0.149	1	195	0.700
Substrate type	0.262 ± 0.207	1.593	1	195	0.208
PC1	0.628 ± 0.293	4.606	1	195	0.033
PC2	0.220 ± 0.284	0.602	1	195	0.439
PC3	−0.329 ± 0.267	1.519	1	195	0.219
PC4	0.044 ± 0.196	0.051	1	195	0.821
PC5	0.175 ± 0.212	0.682	1	195	0.410
PC6	0.072 ± 0.238	0.092	1	195	0.762
(b) *E. nuttallii* area (*F*_9,195_ = 4.412, *p* < 0.001, *r* ^2^ = 0.087)					
Depth	0.002 ± 0.105	0.042	1	195	0.837
*D. polymorpha* presence	0.178 ± 0.090	3.912	1	195	0.049
Substrate type	−0.155 ± 0.092	2.848	1	195	0.093
PC1	0.106 ± 0.089	1.408	1	195	0.237
PC2	−0.080 ± 0.088	0.825	1	195	0.365
PC3	−0.450 ± 0.102	19.619	1	195	<0.001
PC4	0.166 ± 0.089	3.465	1	195	0.064
PC5	0.143 ± 0.089	2.609	1	195	0.108
PC6	−0.007 ± 0.091	0.006	1	195	0.940
(c) *E. nuttallii* proportional cover (*F*_9,195_ = 4.528, *p* < 0.001, *r* ^2^ = 0.206)					
Depth	0.057 ± 0.093	0.378	1	195	0.539
*D. polymorpha* presence	0.061 ± 0.079	0.592	1	195	0.443
Substrate type	−0.298 ± 0.081	13.680	1	195	<0.001
PC1	0.104 ± 0.157	0.439	1	195	0.508
PC2	0.054 ± 0.094	0.324	1	195	0.570
PC3	−0.407 ± 0.089	20.830	1	195	<0.001
PC4	−0.069 ± 0.086	0.641	1	195	0.424
PC5	−0.020 ± 0.079	0.064	1	195	0.801
PC6	0.043 ± 0.080	0.289	1	195	0.592

*Note:* Regression slope (*β*) values are directly comparable. Categorical variables (*D. polymorpha* and substrate type) were converted to 0/1 and standardised; thus *β*‐values represent the slope between the mean values of the dependent variable between each category.

## 3 RESULTS

### 3.1 Distribution

**FIGURE 1:**
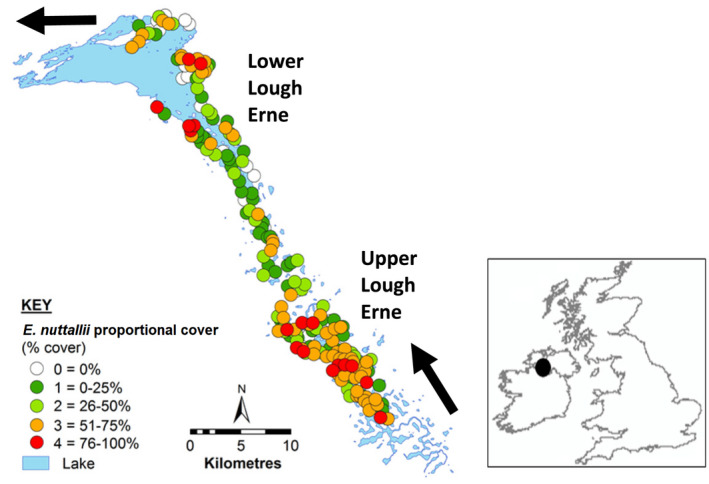
*Elodea nuttallii* patch proportional cover (category intervals of % cover) during 2015 throughout Lough Erne (see insert for location [black dot] within the UK). The arrows show the direction of water flow inflow and outflow

**FIGURE 2:**
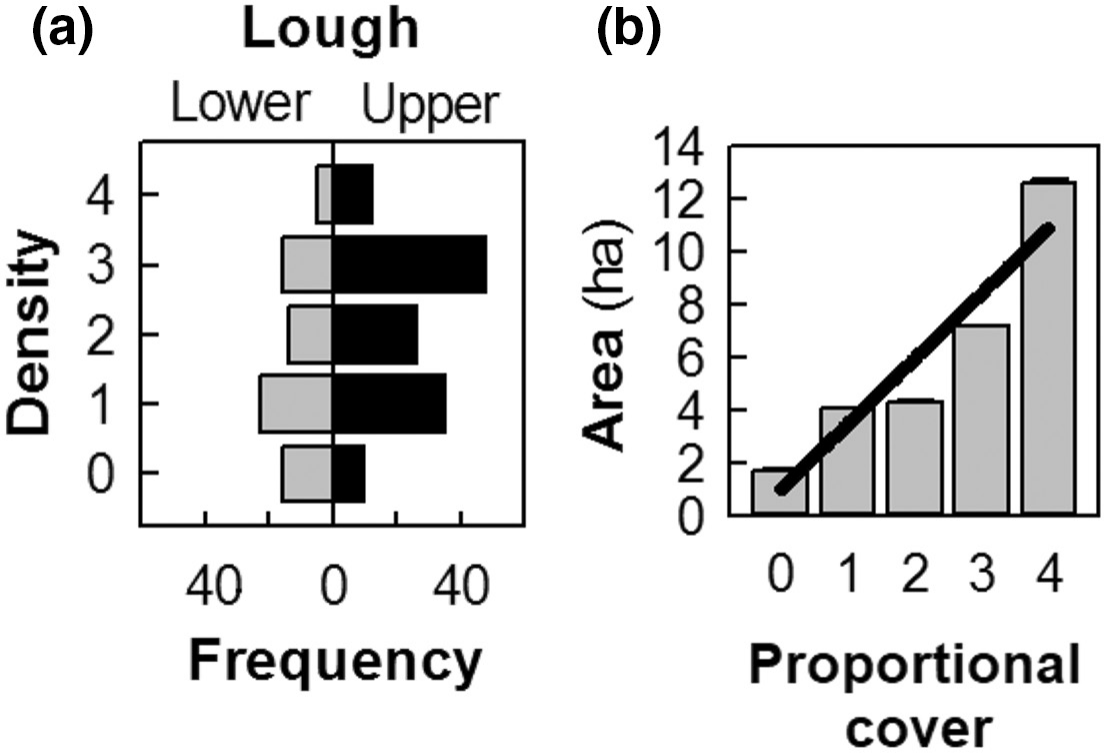
(a) Frequency of *Elodea nuttallii* patches in relation to the proportional cover category for Lower and Upper Lough Erne and (b) relationship between proportional cover and mean patch extent (i.e., area in ha)

*Elodea nuttallii* was found to be widespread throughout the Lough Erne (Figure 1) but occurred most frequently and with greater proportional cover in Upper reaches of the system (Figure 2a). *Elodea nuttallii* proportional cover was positively related to patch extent (i.e., size of area in hectares; Kruskal‐Wallis *χ* ^2^_*df* = 4_ = 33.306, *p* < 0.001; Figure 2b), thereby indicating that larger patches of *E. nuttallii* tended to have a higher dominance by the invader within the community. Patch extent varied from 0.04 to 50.6 ha. Proportional cover category “0” representing 0% *E. nuttallii* and 100% native macrophyte had a mean patch size of ~2 ha. Proportional cover category “1” representing ≤25% *E. nuttallii* cover had a mean patch extent of *~*4 ha; twice that of native macrophyte patches. Patch extent increased with proportional cover, with category ‘4’ (i.e., 75%–100% *E. nuttallii* cover) having a mean patch size of *~*12 ha.

A total of 31 environmental variables were reduced to six principal component axes which captured 81.752% of variation in the dataset (Table 1). Of these, two contributed significantly to subsequent GLMMs: PC1 accounted for 33.150% and PC3 for 13.395% of environmental variation (Table 1). *Elodea nuttallii* occurrence was positively associated with PC1 (Table 2a), while the extent of each patch was negatively associated with PC3 and positively associated with *D. polymorpha* presence (Table 2b). Patch proportional cover was negatively associated with PC3 and varied with substrate type (Table 2c). Not all variables that contributed to PC1 and PC3 had loadings >0.5 (Table 1) with most, but not all, significantly associated with *E. nuttallii* in univariate tests (Table 3).

**TABLE 3:** Univariate tests of the relationship between *Elodea nuttallii* (a) occurrence, (b) extent and (c) proportional cover with the individual explanatory variables making up the principal component axes with which they were related in Table 2

Dependent variable; test statistic	PCA	Independent variable	Statistic	*p*
(a) *E. nuttallii* occurrence	PC1	CHL A	−3.226	**0.001**
Mann–Whitney *Z*		TOxN *trend*	−3.032	**0.002**
		pH	−2.802	**0.005**
		SS	−2.734	**0.006**
		COND	−2.695	**0.007**
		TOxN	−2.562	**0.010**
		P(SOL)	−2.416	**0.016**
		P(SOL) *trend*	−2.228	**0.026**
		P(TOT)	−2.047	**0.043**
		ALK	−2.020	**0.043**
		BOD *trend*	−1.987	**0.047**
		NH_4_ *trend*	−0.238	0.812
		Other woody habitats	−0.180	0.857
(b) *E. nuttallii* extent	PC3	COL	−0.257	**<0.001**
Spearman’s *r*		NH_4_ *trend*	0.179	**0.010**
		BOD	0.160	**0.022**
		NH_4_	−0.159	**0.023**
		Urban land cover	−0.156	**0.026**
(c) *E. nuttallii* proportional cover	PC3	COL	22.821	**<0.001**
Kruskal–Wallis *χ* ^2^		NH_4_ *trend*	17.528	**0.002**
		BOD	15.390	**0.004**
		NH_4_	13.911	**0.008**
		Urban land cover	5.877	0.209

*Note:* The values in the statistic column reflect the test statistic named under the dependent variable.

### 3.2 Occurrence

**FIGURE 3:**
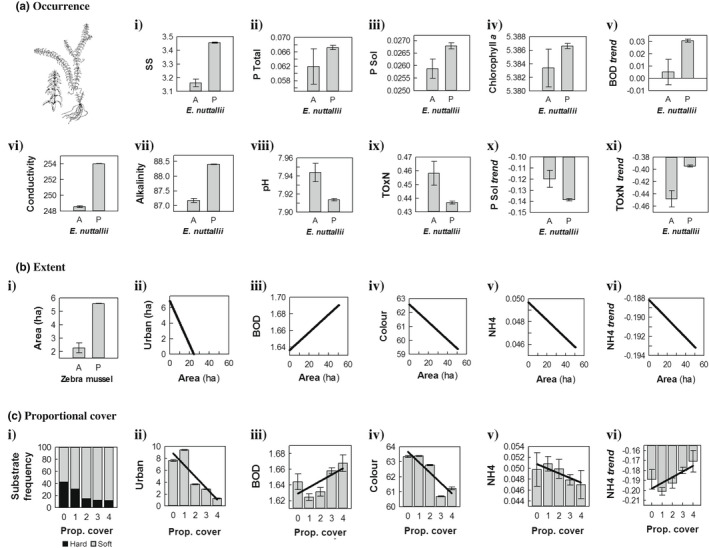
Relationships between *Elodea nuttallii* measured as (a) occurrence (*x*‐axis a = absence, P = presence), (b) extent (i.e., area in ha) and (c) proportional cover (categorical 0 < 1 < 2 < 3 < 4) against water quality variables identified as significant independent correlates of each dependent variable in Table 3. For definitions and units see Table S1 and Figure S1. Values are means ±1 *SE*

Sites invaded by *E. nuttallii* had significantly higher values for suspended solids, phosphorus (P, both total and soluble), chlorophyll‐*a*, conductivity and alkalinity (and thus had lower values for pH) than uninvaded sites (Figure 3a). Patches of *E. nuttallii* were associated with lower total oxidised nitrogen (N) than native macrophyte patches. Values for soluble P and total oxidised N exhibited declines throughout the lake system from 2006 to 2015 (i.e., had negative temporal trends). However, declines of soluble P were greater (i.e., had a steeper slope) and declines of total oxidised N were lower (i.e., had a shallower slope) where *E. nuttallii* had invaded compared to where it was absent. Likewise, presence of *E. nuttallii* accelerated increases in biological oxygen demand (BOD) (Figure 3a).

### 3.3 Extent

*Elodea nuttallii* patches were larger (~5–6 ha) in extent when *D. polymorpha* was present than when it was absent (1–3 ha: Figure 3bi). Of those variables associated with PC3, five exhibited significant relationships with *E. nuttallii* patch extent (Table 3). Landscapes with a high proportion of urban or suburban development had the smallest patches of *E. nuttallii*, whereas the largest patches (>20 ha in size) were found in parts of the lake system that had no such development. No other land cover or land use variables were associated with *E. nuttallii*. Large patches had higher BOD than smaller ones and had lower values for Hazen scale water colour (i.e., the water within large patches was clearer). Ammonium (NH_4_) declined throughout the study system from 2006 to 2015, but values were lower and its decline faster within large *E. nuttallii* patches.

### 3.4 Cover

High proportional cover of *E. nuttallii* was likely to occur on soft rather than hard substrates, whereas native macrophyte patches (proportional cover category “0”) were slightly more likely to occur on the latter (Figure 3c). As for extent, the proportional cover of *E. nuttallii* was greatest in areas with lower levels of urban development, and higher covers were associated with lower water colour (i.e., lower Hazen values) and reduced NH_4_. Greater coverage of *E. nuttallii* intensified BOD, but also was associated with a reduction in the rate of decline of NH_4_ in the study systems at highest cover (proportional cover categories “3” and “4”; Figure 3c).

## 4 DISCUSSION

The present study identified significant abiotic and biotic environmental determinants of the distribution and impacts of the globally widespread, highly invasive, aquatic macrophyte *E. nuttallii*. The presence of *E. nuttallii* was correlated with water chemistry variables and substrate type, and patch size was associated with invasive *D. polymorpha* presence. Using a time series of spatial data from our study system, we also found evidence that *E. nuttallii* has altered key chemical characteristics of the system through time, specifically through accelerating rates of soluble P decline, increasing biological demands for dissolved oxygen, reducing total oxidised N losses, and slight reductions in NH_4_.

The differences in significant principal components indicate that *E. nuttallii* occurrence, extent and proportional cover were each associated with different abiotic and biotic variables, with complex combinations thus influencing invasion status. Proportional cover of patches was significantly related to the areal extent of *E. nuttallii*, such that the larger the area colonised the greater the proportional coverage — as would be expected from expansion from a central inoculum. The positive association between the extent of *E. nuttallii* swards and the presence of invasive *D. polymorpha* empirically corroborated laboratory findings that indicated a mutualism between these species (but not with the benign co‐occurring alien macrophyte *E. canadensis*; Crane et al., 2020). Although it might be thought that mussel beds provide a complex, compact, biogenic reef‐like substrate that offers an anchor for attachment, given that patch proportional cover was associated with soft (not hard) substrates, it seems that the driver of the association between patch proportional cover and *D. polymorpha* may be independent of substrate type and influenced more by environmental correlates, such as water quality variables. Therefore, in an absence of suitable substrates, *D. polymorpha* could use *E. nuttallii* as attachment surfaces, indeed, many of the larger patches of *E. nuttallii* were found in silty, sheltered bays where uprooting forces inhibiting its colonisation would be minimal, whereas native macrophytes were more commonly associated with hard substrates. However, the lack of significant relationship between *E. nuttallii* occurrence/proportional cover and *D. polymorpha* may indicate that this mutualism only effectively promotes extent at later stages of invasion (i.e., after establishment), where it exacerbates rates of spread ‐ and thus aerial extent.

In clear waters (i.e., low turbidity), *E. nuttallii* can maximise growth and extend its range into deeper water (Barko & Smart, 1981; Dar et al., 2014; Maberly & Madsen, 2002; Szabó et al., 2018). The filtering activity of *D. polymorpha* increases this water clarity (Chambers & Kalff, 1985; Higgins et al., 2008), thereby allowing deeper light penetration and potentially increasing the extent over which macrophytes can grow. Furthermore, experimental data indicate that *E. nuttallii* growth is strongly enhanced by the presence of *D. polymorpha* (Crane et al., 2020). Accordingly, when considering the high abundance of *D. polymorpha* in Upper Lough Erne (9.5 billion individuals; 2,238 tonnes; Maguire & Gibson, 2005), where *E. nuttallii* colonisation has been most successful, this mutualism potentially has a major capacity to mediate invasion dynamics at large scales. Reciprocally, there appears to be a mutualistic relationship whereby, in the field, mussels utilise *E. nuttallii* as a settling substrate (Bodamer & Ostrofsky, 2010; Crane et al., 2020; Horvath & Lamberti, 1997; MacIsaac, 1996). As such, mussel recruitment, coupled with macrophyte growth facilitation, may represent a positive feedback cycle that drives invasion success between the two groups.

The presence of *D. polymorpha* significantly influenced the extent of *E. nuttallii*, yet other environmental correlates were potentially determinants of macrophyte invasion dynamics. Conductivity and alkalinity were significantly higher at sites invaded by *E. nuttallii* than in uninvaded sites. *Elodea nuttallii* was, however, present where pH was lowest, preferring near‐neutral conditions. This is consistent with the results of Jones et al. (2000), who found that *E. nuttallii* photosynthesis is reduced at >pH 7, in part due to low availability of CO_2_ under alkaline conditions. Sites invaded by *E. nuttallii* also had significantly higher values for suspended solids. A possible explanation for this relationship might be that *E. nuttallii* was most commonly found on soft (i.e., silty) substrates, where suspended particles can be dislodged within dense mats of macrophytes. However, a more detailed assessment of substrate types in relation to grain size and composition should be considered, rather than the straightforward visual estimation of substrate used in the present study. Nevertheless, sites invaded by *E. nuttallii* had significantly higher values for total and soluble phosphorus. *Elodea* species require moderate‐to‐high nutrient levels within an ecosystem, and as such prefer eutrophic waters (Baldy et al., 2015; Melzer, 1999). The P content in the sediment can be taken up via *E. nuttallii* roots and released back into the water column via leakage from the plant or decomposition (Barko & Smart, 1980; Carignan & Kalff, 1980; van Donk et al., 1993), which would in turn support higher values of P within *E. nuttallii* patches and increase growth rates. This increased growth may afford *E. nuttallii* a competitive advantage in canopy formation, thus inhibiting competition from co‐occurring species such as *E. canadensis* (Kelly et al., 2015). If plant biomass is high, there also can be significant effects on P and N cycling (Frankouich et al., 2006; James et al., 1999; Ozimek et al., 1993). Indeed, values for soluble P declined throughout the lake system over the study period, and particularly in the presence of *E. nuttallii*. Nonetheless, the importance of pollution regulation measures should not be discounted for the general decline in P from agricultural runoff, and *E. nuttallii* appeared to perform better in rural, agricultural areas as compared to those with greater urbanisation.

Ammonium in the system also declined over the study period, and was lower, and its decline faster, within large *E. nuttallii* patches. Equally, total oxidised N was lower in *E. nuttallii* patches, but declined more slowly, when *E. nuttallii* was present. *Elodea nuttallii* takes up NH_4_‐N to generate plant biomass (Bishop & Eighmy, 1989; Dendéne et al., 1993; Rolland & Trémolières, 1995), and biomass increases as nutrient concentration rises (Rolland & Trémolières, 1995). Therefore, the lower values and greater temporal declines in NH_4_ could be attributed to uptake by *E. nuttallii* during expansion and colonisation. Furthermore, a laboratory study has shown that where *E. nuttallii* is present with high *D. polymorpha* densities there is a greater depletion of NH_4_ than in the absence of *D. polymorpha* (Crane et al., 2020), even though *D. polymorpha* can excrete significant concentrations of NH_4_ (Gardner et al., 1995). This suggests that NH_4_ uptake by *E. nuttallii* increases at higher *D. polymorpha* densities, and thus their co‐occurrence in the present study may have synergistically altered such nutrient levels. Nevertheless, these data were generated from spatially kriged (so called “rubber‐sheeted”) values between recording stations throughout the lake system. The average distance between recording stations was 5 km whilst the average distance between *E. nuttallii* patches was only 0.35 km. Spatially kriged values from heat maps extracted at a higher spatial resolution than that of the input data, derived from interpolated and extrapolated averages across space, are likely to produce small absolute differences as a result of how they were generated. This should not be interpreted as rendering the relationships established here as less meaningful, but rather support the found relationships probably being indicative of greater biological and ecological significance if only real‐time values had been available for each patch of *E. nuttallii* synchronously with the survey, which was not just impractical but impossible. It also should be noted that this study surveyed mature plants only and that immature plants may have occurred at the study sites at some depth (thus, being missed by surveys). If sufficiently numerous, these immature plants also may have impacted water chemistry variables.

Sites invaded by *E. nuttallii* additionally had significantly higher values for chlorophyll‐*a*, indicative of higher productivity, most notably of epiphytic algae associated with *E. nuttallii* (Kelly et al., 2015). Like the increased amounts of suspended solids within dense *E. nuttallii* patches, the reduced surface water turbidity where *E. nuttallii* patches reach the water surface may provide suitable conditions for rapid, localised algal growth. In turn, sites invaded by *E. nuttallii* and *D. polymorpha* had significantly higher values for BOD than uninvaded sites, and the larger the patch of *E. nuttallii*, the greater the oxygen demand. The BOD generally increased throughout the system over the duration of the study period. The vast volumes of weed occurring in the lake will, cyclically, die and decay, a process which utilises oxygen (Asaeda et al., 2001; Jewell, 1971; Pereira et al., 1994). Concerningly, the lowering of dissolved oxygen can impact other aquatic life and potentially affect the species composition, having significant knock‐on effects on the biological functioning of shallow, freshwater systems (Buscemi, 1958; Dickey et al., 2021).

From a management perspective, although the prevention of further invader spread generally is considered to be the most efficient management approach (Coughlan et al., 2020), an improved understanding of how invasive macrophytes interact with the abiotic and biotic components of ecosystems can aid decision makers in prioritising the allocation of resources for ongoing control of populations of invasive macrophytes. A broad range of management options have been trialled for *E. nuttallii* populations, including biological control with herbivorous fishes, mechanical control via cutting, shading and water drawdown, as well as chemical control via herbicide applications (Zehnsdorf et al., 2015). Effective methods for the prevention of introduction and further spread of invasive macrophytes, such as thermal shock (e.g., steam; Crane et al., 2019), can mitigate ongoing ecological and socioeconomic impacts that arise from long‐term post‐invasion management (Cuthbert, Pattison, et al. 2021a; Cuthbert, Diagne, et al. 2021b; Zehnsdorf et al., 2015). In addition, an understanding of environmental determinants of invasion success can help to pinpoint resources for preventative measures for invasive macrophytes, such as via biosecurity (Coughlan et al., 2020). Likewise, careful management of existing populations of invasive macrophytes could lead to improved water quality, such as through the removal of excess P. Accordingly, future manipulative studies should be carried out to complement the observational fieldwork to assess abiotic and biotic correlates with the occurrence, extent and cover for *E. nuttallii*, as well as other invasive macrophytes to enable strategic management practices. Such approaches could include controlled environmental studies to determine the direct and interacting effects of environmental variables found to influence invasion observations here and in other regions. In particular, an improved understanding of interactive effects between bivalves and macrophytes under a range different abiotic conditions is needed (Crane et al., 2020). To achieve this, basic laboratory mesocosm experiments can be scaled to the level of infestation observed at an invaded site (Coughlan et al., 2021), which will support cost–benefit assessments for management interventions. In addition, this paper presents data from surveys from a single system collected during summer months. To confirm that our findings are not specific to Lough Erne, studies incorporating a range of locations and seasonal assessments also should be conducted.

## 5 CONCLUSIONS

We show that invasion of freshwater lakes by *E. nuttallii* is associated with a range of water quality variables, their change over time, the influence of surrounding land cover and use, as well as mutualism with other invasive species. It is evident that many abiotic and biotic factors influence the distribution and abundance of *E. nuttallii*. Accordingly, it is necessary to consider the total environment when planning management strategies. Increased nutrients have a considerable influence on the growth of *E. nuttallii,* therefore, reductions in these would be beneficial. *Elodea nuttallii* was positively associated with more rural locations with lower urbanisation, perhaps suggesting that it can consolidate its growth in locations less disturbed by river activities including boating or by utilising the excess nutrient runoff from nearby farmland. Within this system, the removal of excess *E. nuttallii* biomass, with its accumulated P, could be a helpful alternative technique to other eutrophication control strategies. Improved management of facilitative invaders such as *D. polymorpha* also could be beneficial. Nevertheless, further assessments are required to elucidate the mechanisms that underpin invasion dynamics and nutrient cycling amongst these freshwater invaders.

## AUTHOR CONTRIBUTIONS

Conceptualisation: KC, AR, HJM, JTAD, NR. Developing methods: KC, LK, NEC, RNC, JTAD, NR. Conducting the research: KC, NR. Data analysis: KC, NR. Preparation of figures and tables: KC, LK, NEC, RNC, NR. Data interpretation: KC, LK, NEC, RNC, NR. Writing: KC, LK, NEC, RNC, AR, HJM, JTAD, NR. Manuscript revision: KC, LK, NEC, RNC, NR. All authors give final approval for publication of this study. All authors agree to act as guarantors for the accuracy and integrity of this study.

## Supporting information


Table S1

Figure S1

## Data Availability

The data are available from the authors upon reasonable request.
