# Supplementary material for: Abiotic and biotic correlates of the occurrence, extent and cover of invasive aquatic Elodea nuttallii
Source: Freshw Biol. 2022 Jul 1;67(9):1559–70. doi: 10.1111/fwb.13960 (PMC9545499; doi:10.1111/fwb.13960)
Supplement: Supplementary file 1 — Table S1 [file FWB-67-1559-s002.docx]

**Supplementary Material**

**Table S1.** Water quality and land cover variables and a description of each. Water quality variables were obtained from the Northern Ireland Environment Agency (NIEA) Water Management Unit. Habitat was CORINE downloaded from the European Environment Agency (EEA).

| **Variable** | **Code** | **Description** |
| --- | --- | --- |
|  |  |  |
| **Water quality** | | |
| pH | pH | Acidity or alkalinity measured using a handheld YSI Digital Professional series field meter |
| Alkalinity | ALK | Samples titrated with standard acid solution and instrumental detection of the end point at pH 4.5 |
| Conductivity | COND | Conductance of a standardised volume of water with 1cm^2^ cross-sectional area and electrodes spaced 1cm apart measured using a Thermometer and probe |
| Colour | COL | Spectrophotometric colorimetery expressed in the 'Hazen' standard unit |
| Suspended Solids | SS | Gravimetry using vacuum filtration to measure small solid particles in suspension as a colloid |
| Soluble Phosphorus | P(SOL) | Concentration of orthophosphate (PO_43-_) captured by colorimetry using a Seal QuAAtro segmented flow system consisting of an auto-sampler, peristaltic pump, chemistry manifold and detector after the sample was digested using persulphate |
| Total Phosphorus | P(TOT) | Same as P(SOL) but capturing the sum of all phosphorus compounds i.e. orthophosphate (PO_43-_) plus organic compounds |
| Ammonia | NH_4_ | Concentration of NH_4_ measured by colorimetry using a Seal QuAAtro segmented flow system consisting of an auto-sampler, peristaltic pump, chemistry manifold and detector |
| Nitrite | NO_2_ | Colorimetry using a Seal QuAAtro segmented flow system consisting of an auto-sampler, peristaltic pump, chemistry manifold and detector to capture nitrite (NO_2_) |
| Nitrate | NO_3_ | Same as NO_2__N but capturing nitrate |
| Total Oxidised Nitrogen | TOxN | Colorimetry using a Seal QuAAtro segmented flow system consisting of an auto-sampler, peristaltic pump, chemistry manifold and detector to measure of nitrite plus nitrate |
| Chlorophyll-*a* | CHLR A | Water samples were filtered, Chlorophyll-*a* extracted from filter paper into a methanol solution read at two wavelengths using an ultraviolet/visible spectrophotometer |
| Dissolved Oxygen | DO | Measured *in-situ* using a YSI Digital Professional series field meter expressed as mg/l |
| Biological Oxygen Demand | BOD | The mass of DO consumed during a 5 day incubation period at 20°C by biochemical oxidation of organic and/or inorganic matter measured using a Hach optical probes expressed as O2 mg/litre |
|  |  |  |
| **Land cover / use** |  | Percentage of 500m buffer that was: |
| Other farmland |  | Arable plus complex cultivation patterns |
| Improved grassland |  | Pastures |
| Conifer plantations |  | Non-native coniferous forest |
| Deciduous woodland |  | Broad-leaved forest plus mixed forest |
| Other woody habitats |  | Transitional woodland scrub |
| Bog, fen & moor |  | Peatbogs, inland marsh, moors and heatlands |
| Urban/suburban areas |  | Continuous and discontinuous urban fabric |
