# Supplementary material for: Abiotic and biotic correlates of the occurrence, extent and cover of invasive aquatic Elodea nuttallii
Source: Freshw Biol. 2022 Jul 1;67(9):1559–70. doi: 10.1111/fwb.13960 (PMC9545499; doi:10.1111/fwb.13960)

Fig. S1. Spatial patterns in mean values of water quality variables throughout Lough Erne (left column) and temporal patterns in water quality variables (i.e. standardised linear regression slope or β value from 2006-2015. Data were collected at measurement stations shown as black dots and missing data were interpolated by Kriging using the Spatial Analyst extension for ArcGIS 10.5.

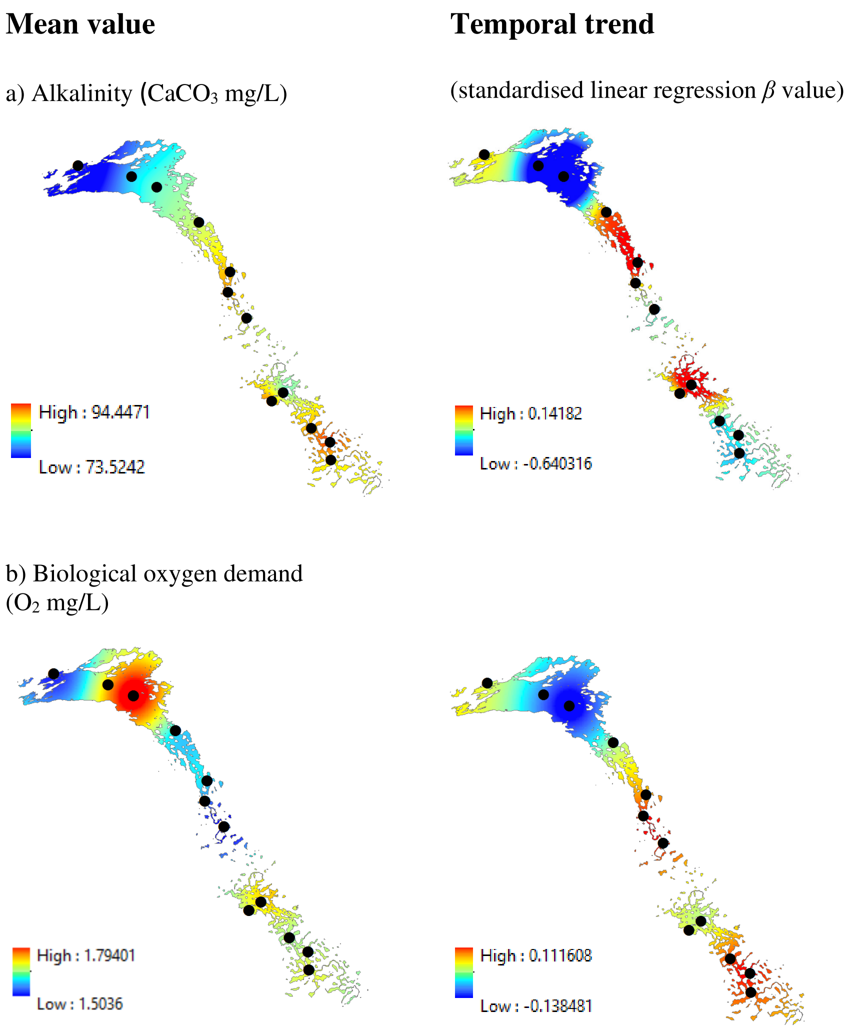

Mean value **Temporal trend**

c) Chlorophyll A (mg/L)

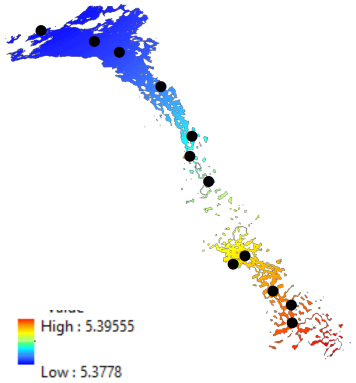

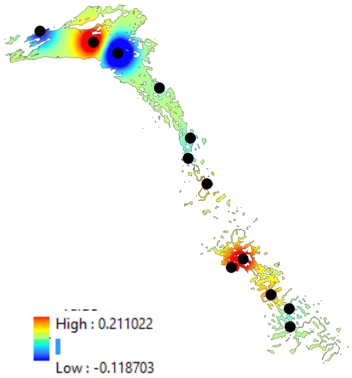

d) Colour (Hazen units: HU)

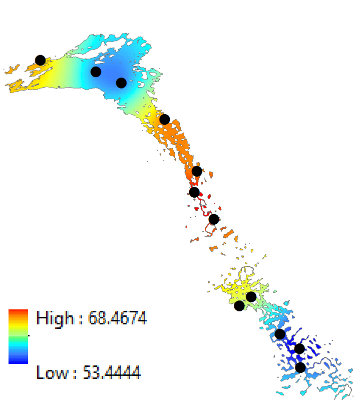

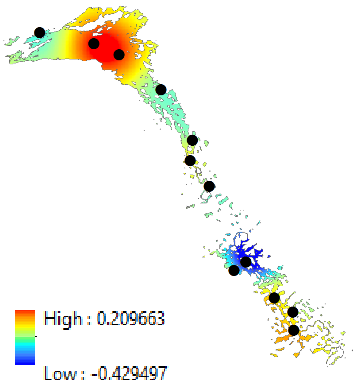

e) Conductivity (µmhos/cm)

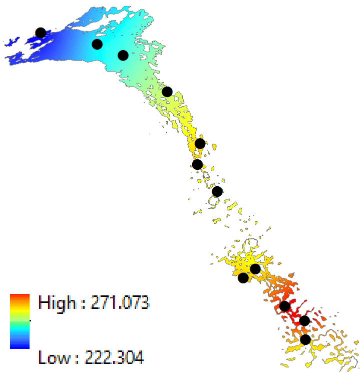

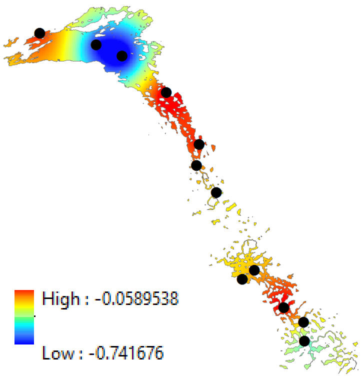

Mean value **Temporal trend**

f) Nitrate (NO_3-N mg/L)

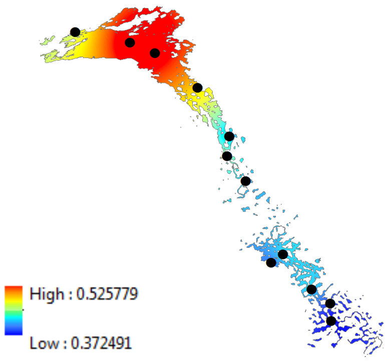

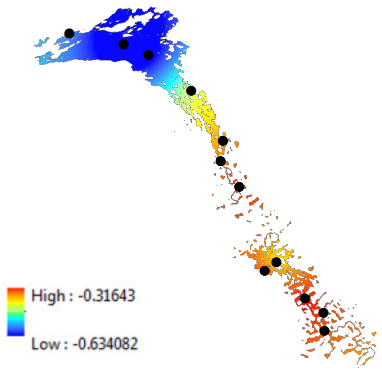

g) Nitrite (NO2-N mg/L)

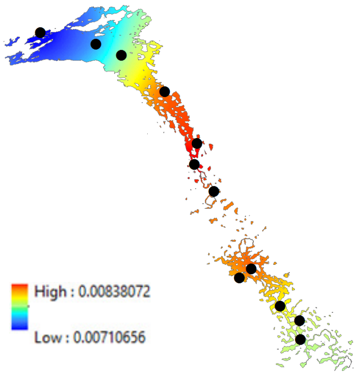

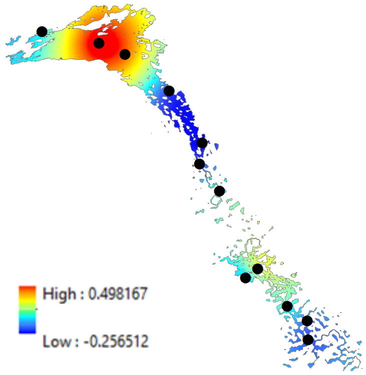

h) Nitrogen (Ammoniacal) (NH4-N mg/L)

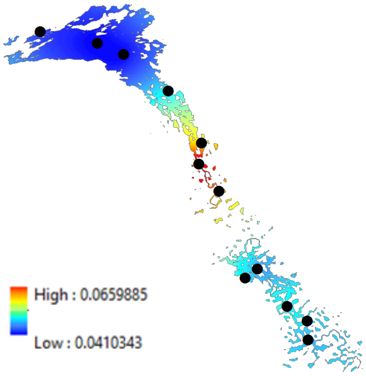

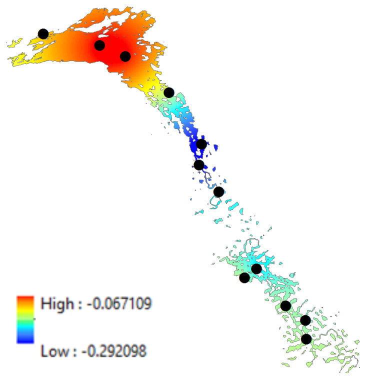

Mean value **Temporal trend**

i) pH (1 - 14)

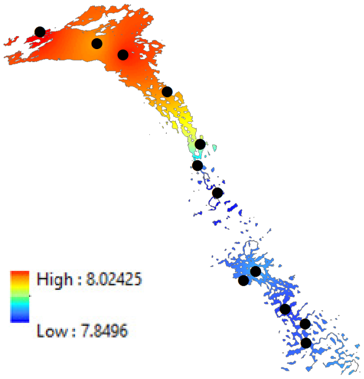

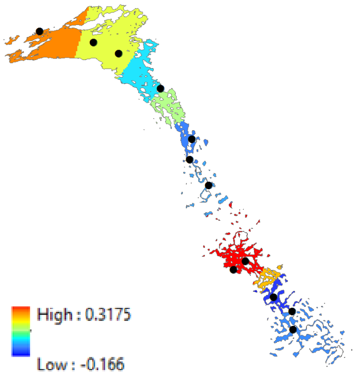

j) Phosphorus soluble (P Sol mg/L)

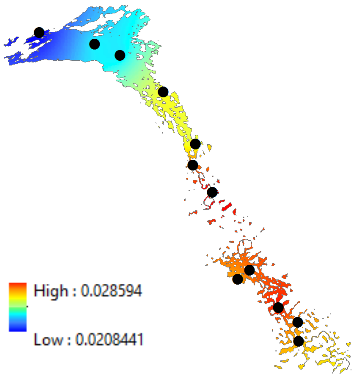

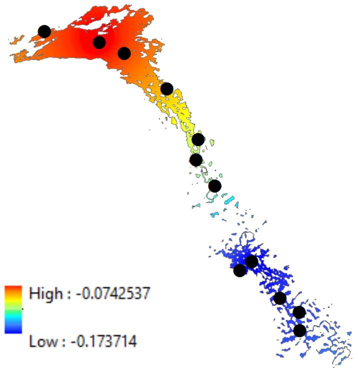

k) Total Phosphorus (P TOT mg/L)

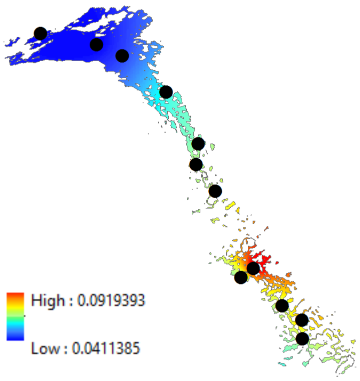

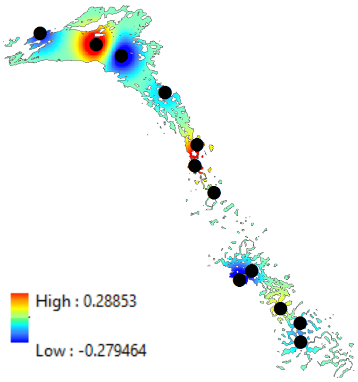

Mean value **Temporal trend**

l) Suspended Solids (mg/L)

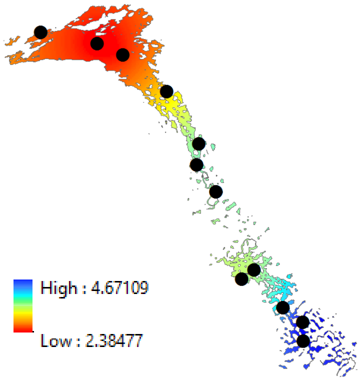

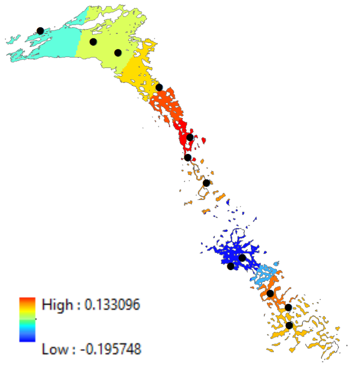

m) Total oxidised nitrogen (TOxN mg/L)

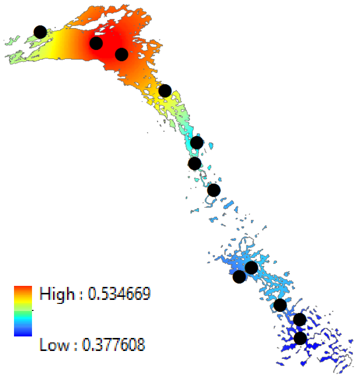

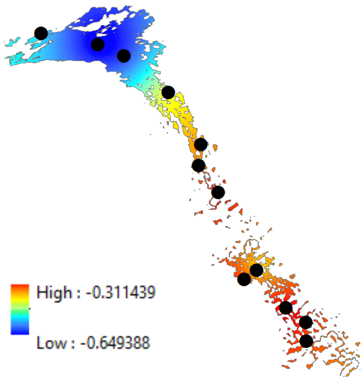

Supplement: Supplementary file 2 — Figure S1 [file FWB-67-1559-s001.pdf]
